# Data Fusion Approaches for the Characterization of Musts and Wines Based on Biogenic Amine and Elemental Composition

**DOI:** 10.3390/s22062132

**Published:** 2022-03-09

**Authors:** Aina Mir-Cerdà, Biel Granell, Anaïs Izquierdo-Llopart, Àngels Sahuquillo, José Fermín López-Sánchez, Javier Saurina, Sonia Sentellas

**Affiliations:** 1 Department of Chemical Engineering and Analytical Chemistry, University of Barcelona, Martí i Franquès 1-11, E08028 Barcelona, Spain; ainamir17@gmail.com (A.M.-C.); biel.granell@gmail.com (B.G.); anais.izquierdo.llopart@gmail.com (A.I.-L.); angels.sahuquillo@ub.edu (À.S.); fermin.lopez@ub.edu (J.F.L.-S.); sonia.sentellas@ub.edu (S.S.); 2 Research Institute in Food Nutrition and Food Safety, University of Barcelona, Av. Prat de la Riba 171, Edifici Recerca (Gaudí), E08921 Santa Coloma de Gramenet, Spain; 3 Serra Húnter Lecturer, Generalitat de Catalunya, E08007 Barcelona, Spain

**Keywords:** sparkling wine, biogenic amines, elemental composition, wine quality, winemaking practices, principal component analysis, data fusion approach

## Abstract

Samples from various winemaking stages of the production of sparkling wines using different grape varieties were characterized based on the profile of biogenic amines (BAs) and the elemental composition. Liquid chromatography with fluorescence detection (HPLC-FLD) combined with precolumn derivatization with dansyl chloride was used to quantify BAs, while inductively coupled plasma (ICP) techniques were applied to determine a wide range of elements. Musts, base wines, and sparkling wines were analyzed accordingly, and the resulting data were subjected to further chemometric studies to try to extract information on oenological practices, product quality, and varieties. Although good descriptive models were obtained when considering each type of data separately, the performance of data fusion approaches was assessed as well. In this regard, low-level and mid-level approaches were evaluated, and from the results, it was concluded that more comprehensive models can be obtained when joining data of different natures.

## 1. Introduction

Data fusion approaches have been considered an excellent way to enrich datasets, particularly for improving the descriptive performance of the method and the overall quality of the information [1,2]. The original datasets obtained with different analytical methods can be simply joined in a global matrix according to so-called low-level data augmentation to be further analyzed with the arsenal of chemometric methods available for characterization, classification, and quantification purposes. In addition, raw data from various sources can be pretreated specifically using chemometric methods, and the resulting individual post-processed matrices can be combined by using mid- or high-level data fusion. Hence, data augmentation approaches are especially fruitful in food analysis for dealing with characterization, classification, and authentication issues [3,4,5].

More specifically, in the case of wines and related alcoholic beverages, several studies have been conducted based on data fusion. Ranaweera and coworkers published an overview of wine authentication based on spectroscopic data and further chemometric analysis [6]. In a broader sense, Arslan and coworkers reviewed the characterization and authentication of alcoholic beverages by nondestructive instrumental techniques and chemometrics [7], and da Costa et al. focused on beers [8]. Among the analytical techniques used to generate data of high quality, near- and mid-infrared spectroscopies have been widely used for the authentication of grappa and other spirits [9,10], wine vinegar [11], Mexican tequila [12], and beers [13,14]. In addition to vibrational data, other spectroscopies, such as UV–vis, excitation–emission fluorescence, and nuclear magnetic resonance, have also been suggested for different classification and characterization purposes [10,11,12,13,14,15,16]. For fast, selective, and sensitive analyses, high-resolution mass spectrometry combined with flow injection analysis has offered new opportunities for red wine discrimination and classification [17]. Electronic devices, including e-noses and e-tongues, have also been introduced for wine characterization, and the data gained from these techniques have often been combined to obtain more comprehensive descriptions [18,19,20,21]. For instance, colorimetric sensor arrays based on dyes exhibiting different cross-selectivities towards the analytes were used for the discrimination of alcoholic beverages, including beers and spirits [22,23,24]. These devices, when operated in the gas phase, resulted in optoelectronic noses in which sample recognition relied on volatile species such as aldehydes and ketones. An interesting review of optoelectronic noses can be found elsewhere [25]. Another widely used approach to generate instrumental data to assess wine features relies on separation techniques, including capillary electrophoresis, gas chromatography, and especially liquid chromatography. Several studies using chromatographic data have been reported in the scientific literature for the classification of Lambrusco wines [26], grappa spirits [10], and rums [27]. Multi-sensor data integrating information from a wide variety of analytical techniques have also been introduced for wine analysis. For instance, Izquierdo-Llopart et al. studied the classification of sparkling wines as a function of grape variety and coupage using concentrations of organic acids, phenolic compounds, antioxidant capacity, pH, total acidity, ethanol, or reducing sugars [28]. In another recent publication, Cavdaroglu and Ozed developed a strategy for the prediction of vinegar processing parameters based on UV–visible and mid-infrared spectra, pH, Brix, total acidity, total flavonoid content, total and individual phenolic contents, organic acid, sugar, and ethanol concentrations [29].

In this publication, we explore the combination of biogenic amine (BA) and elemental compositions in an attempt to find markers of winemaking practices and product quality. BAs are low-molecular-weight nitrogenous compounds arising from the decarboxylation of amino acids, which are especially abundant in wine, cheese, meat, fish, and spoiled products [30]. BAs provide valuable information on wine quality and oenological factors [31,32]. In wine and cava, concentrations of BAs can provide valuable information on product quality, as they are also a good indicator of food safety. BA contents depend on the agricultural practices involved in the production of grapes, the grape variety used, and the processes of vinification and aging, especially if the wine is exposed to the activity of microorganisms or free amino acids are present. Indeed, alcoholic and especially malolactic fermentations are principal processes involved in BA generation due to the presence of yeasts and bacteria. Some microorganisms can decarboxylate amino acids with specific enzymes to form biogenic amines, thus significantly increasing their content. Once the BAs are formed, they are relatively stable in the wine samples. Putrescine, ethanolamine, tyramine, and histamine are abundant in the final products to be commercialized. Some representative recent examples dealing with the relevance of BAs as biomarkers of wine quality are summarized in Table 1.

The elemental composition has also been exploited to characterize and authenticate wines as a function of geographical, varietal, and oenological factors [41,42,43]. For instance, elements such as Al, B, Ca, Cu, Fe, K, Mg, and Mn are relevant markers of some wine features. Despite the apparent disparity between the two types of analytes, they are used to characterize the quality of food products, particularly wines, thus providing complementary information from inorganic and organic species. In addition to the simple combination of BA and elemental profiles in a sample-wise augmented data matrix, other data fusion approaches are explored.

## 2. Materials and Methods

### 2.1. Chemicals and Solutions

Unless otherwise specified, all reagents used were of analytical grade. General reagents for biogenic amine profiling and elemental analysis were as follows: nitric acid (65% (*w*/*w*), PanReac ApplyChem, Castellar del Vallès, Spain), sodium tetraborate (Merck KGaA, Darmstadt, Germany), dansyl chloride (98%, Thermo Fisher Scientific, Waltham, MA, USA), formic acid (>96%, Merck), acetonitrile (UHPLC PAI-ACS SuperGradient, PanReac), and chloroform (≥99.8%, Fluka, Buchs, Switzerland). Purified water was generated with an Elix3 system (Millipore, Bedford, MA, USA). Solutions for BA derivatization were dansyl-Cl reagent, prepared at a concentration of 10 mg mL^−1^ in acetone, and 0.1 mol L^−1^ sodium tetraborate as the buffer solution (pH 9.2). For ICP-OES and ICP-MS, samples were diluted with 1% (*v*/*v*) HNO_3_.

Biogenic amine standards were as follows: 1,5-diaminopentane (cadaverine, 98%), 1,4-diaminobutane dihydrochloride (putrescine, 99%), spermidine trihydrochloride (99%), and spermine tetrahydrochloride (99%) from Alfa Aesar (Kandel, Germany); histamine hydrochloride (≥99%), 2-phenylethylamine hydrochloride (≥99%), tryptamine hydrochloride (≥98%), tyramine hydrochloride (≥97%), octopamine hydrochloride (≥99%), and agmatine sulfate (≥99%) from Fluka (Buchs, Switzerland); ethanolamine hydrochloride (≥98%) and hexylamine (≥98%) from TCI (Tokyo, Japan), the latter of which was used as the internal standard. Each amine was prepared as a 1000 mg L^−1^ stock solution in the laboratory by dissolution in Milli-Q water. Calibration standards were prepared by appropriately diluting stock solutions in a range from 0.1 to 50 mg L^−1^. Stock and working solutions were stored at 4 °C until use.

Certified ICP grade single-element standards of 1000 mg L^−1^ in 1% (*v*/*v*) HNO_3_ were purchased from Inorganic Ventures (Christiansburg, VA, USA). Calibration standards for ICP-MS and ICP-OES measurements were prepared by carrying out the required dilutions with 1% (*v*/*v*) HNO_3_.

### 2.2. Samples

Musts, wines, and sparkling wines were kindly provided by Codorníu SA (Sant Sadurní d’Anoia, Spain). Table 2 describes the set of 20 monovarietal products from Xarel·lo grapes and 20 monovarietal products from Pinot Noir grapes, produced in Penedès and Costers del Segre regions (Catalonia, Spain). Each grape variety had five oenological classes, comprising musts, base wines, stabilized wines, 3-month-aged sparkling wines, and 7-month-aged sparkling wines, and four quality levels were defined for each class: A, B, C, and D, where A is the top quality and D is the lowest one. A more detailed description of the quality features is given elsewhere [33]. Sample coding is detailed in Table 2.

A quality control (QC) sample was prepared to assess the reproducibility of the analytical methods and the significance of the PCA models by mixing 1 mL of each must/wine/cava sample.

### 2.3. Instruments

The chromatographic system consisted of an Agilent 1100 Series HPLC instrument from Agilent Technologies (Waldbronn, Germany), with degasser (G1379A), binary pump (G1312A), automatic injector (G1392A), diode-array UV–vis detector (G1315B), and fluorescence detector (FLD, G1321A). Instruments for elemental analysis were Optima 3200RL ICP-OES and Nexlon 350D ICP-MS spectrometers (both from Perkin Elmer, Waltham, MA, USA) equipped with Ar plasma. Rh was used as the internal standard in ICP-MS.

### 2.4. Analytical Procedures

#### 2.4.1. Biogenic Amine Determination

The method for the determination of BAs based on offline derivatization and liquid chromatography with fluorescence detection was established and validated elsewhere [33]. Briefly, BAs were derivatized offline by mixing 250 µL of sample (or biogenic amine standard), 250 µL of dansyl-Cl reagent solution, and 250 µL of buffer solution (pH 9.2). The reaction was developed at 40 °C in a thermostatic water bath (Tectron 473-100, J.P. Selecta, Barcelona, Spain) for 60 min. Derivatives were further extracted by adding 750 µL of chloroform and applying mechanical shaking for 10 min (Vortex 3 IKA, Staufen, Germany). The organic fraction was evaporated to dryness and redissolved in 600 µL of acetonitrile/water (50:50, *v*/*v*).

Derivatized samples were analyzed by HPLC-UV-FLD using a core–shell column (Kinetex C18, 150 mm × 4.6 mm I.D., 2.6 µm particle size) from Phenomenex (Torrance, CA, USA). A 0.1% (*v*/*v*) formic acid aqueous solution and acetonitrile (ACN) were used as the mobile phase components under an elution gradient program based on increasing the percentage of ACN (see Reference [33]). The flow rate was 0.7 mL min^−1^, and the injection volume was 10 µL. UV detection was at 254 nm, and FLD was at 320 nm for excitation and 523 nm for emission.

Samples were processed in triplicate and analyzed randomly, injecting the QC sample every 10 samples.

#### 2.4.2. Elemental Composition Determination

Samples diluted at a 1/10 ratio with 1% HNO_3_ were directly analyzed by ICP-OES and ICP-MS, as explained elsewhere [43]. A blank solution (1% HNO_3_) and the quality control (QC) sample were analyzed every 15 samples to check for cross-contamination and assess the repeatability of the results. All samples were analyzed in triplicate.

### 2.5. Data Analysis

ANOVA was performed with Microsoft Excel (Microsoft, Redmon WA, USA), and multivariate data analysis was conducted with SOLO software (Eigenvector Research, Inc. Manson, WA, USA).

Principal component analysis was applied for an exploratory characterization of musts, wines, and sparkling wines to try to identify patterns of oenological steps, product quality, and varieties using compositional data as the source of analytical information. Further details on PCA and other chemometric methods can be found elsewhere [44,45].

For each instrumental technique, data matrices (X-matrices) were generated, in which each row corresponded to a sample replicate and each column corresponded to a given analyte. X-matrix dimensions were 120 × 11 for BAs and 120 × 38 for the elemental composition. Hence, the low-level data fusion matrix was 120 × 49. The individual matrices of BA and elemental composition were pretreated by PCA to extract nine PCs, which were further combined in the mid-level approach, thus resulting in an augmented matrix of 120 × 18.

## 3. Results and Discussion

The performance of BA profiles and elemental composition as a source of potential descriptors of must, base wine, stabilized wine, and sparkling wine was previously assessed by Mir-Cerdà et al. and Granell et al., respectively [33,43]. In those studies, the two datasets were studied separately, so independent conclusions were drawn. In this work, however, we wanted to combine information from the two compositional profiles in order to try to improve the quality of the description and provide more comprehensive knowledge of sample features and the influence of the oenological practice, grape variety, and product quality. Hence, data matrices generated in the previous studies were fused using low- and mid-level approaches for further chemometric analysis.

First, we present the most important results extracted from the previous studies to highlight the outcomes of their independent chemometric characterizations of must, wine, and sparkling wine samples. As indicated in Section 2 (Materials and Methods), ethanolamine, putrescine, tyramine, histamine, cadaverine, spermine, spermidine, tryptamine, octopamine, lysine, and phenylethylamine were quantified by the HPLC-FLD method, and up to 36 elements were determined by ICP-OES or ICP-MS (Al, As, B, Ba, Ca, Cd, Ce, Co, Cr, Cs, Cu, Fe, Ga, K, La, Li, Mg, Mn, Mo, Na, Nd, Ni, P, Pb, Rb, S, Sb, Sc, Si, Sn, Sr, Ti, U, V, W, Y, Zn, and Zr). Table 3 shows the concentrations of some of the most significant components, which were selected because of their relevance as potential descriptors of sample type or sample quality features. As can be seen, potassium is overexpressed in musts, putrescine and histamine levels are increased in samples of C or D quality, sulfur is remarkably higher in base and stabilized wines, and ethanolamine and sodium concentration are, in general, slightly higher in sparkling wines. Similar patterns can be observed for other compounds.

Quantification errors were estimated according to Mir-Cerdà and Granell (see References [33,43]) from the analysis of the QC sample with the respective methods. The quantification errors of putrescine and ethanolamine (the most abundant amines) were 3.4% and 3.2%, respectively. For the other compounds, errors were below 10% (e.g., 5.5 for agmatine, 8.4 for tryptamine, 9.0 for phenylethylamine, 7.8 for cadaverine, 9.7 for histamine, and 6.6 for tyramine), except for spermidine and spermine (ca. 25%, caused by higher derivatization and stability issues). For the elemental composition, the errors in ICP-OES values of metals occurring at concentrations of the order of magnitude of 1 mg L^−1^ were lower than 2% (e.g., 0.9 for Mg, 1.2 for Ca, 1.5 for P, and 1.7 for Na), except for K (3.5%). For other important descriptors determined by ICP-MS, errors were ca. 5% (e.g., 4.3 for Fe, 2.7 for B, 6.3 for Cs, 7.3 for S, 6.4 for Sr, 5.6 for Ba, 5.0 for Mn, 4.5 for Cu, and 5.7 for Al). Trace elements occurring at concentrations between 1 and 100 µg L^−1^ showed errors ranging from 7 to 20% (e.g., Li, Mo, Ni, Zn, and V). The descriptive performance of these elements was more limited, thus mainly contributing to the noise.

In the case of BAs, PCA models showed well-defined clusters for each sample type, and the loading plot highlighted putrescine and ethanolamine as the best descriptors of the winemaking process. It was found that concentrations in must samples were, in general, low (except for ethanolamine). A remarkable rise in BAs was observed at the base wine stage, i.e., after the first alcoholic fermentation. This increase was even more dramatic for wines subjected to malolactic fermentation since this process has been identified as a major factor in the generation of BAs. After this stage, BA levels remained constant or slightly decreased with stabilization, second fermentation, and aging. This pattern was also observed for other amines, such as tyramine, histamine, and cadaverine. Regarding product quality, differences among high- and low-quality products were noticeable.

Regarding elemental composition, interesting patterns in the evolution of the composition of elements such as K, Cu, Ca, S, and Mg during the vinification process were found. Furthermore, some elements were recognized as potential markers of product quality. For instance, the top-quality (A) samples displayed lower contents of some elements, such as K and Ca. Other elements such as Mg, Mn, Na, Ni, Sr, and Zn also appeared in higher levels in C and D products since they were introduced from additives used in different technological processes. PCA showed two separated clusters corresponding to musts and fermented samples, thus confirming the noticeable differences due to the addition of several substances during fermentation, clarification, and stabilization processes. Subsequently, during the aging process, they tended to precipitate together with the lees, so their concentrations typically decreased in aged products. 

Given the remarkable conclusions preliminarily extracted from the analysis of BA and elemental composition datasets, in the following analysis, we aimed to assess the performance of the combination of the two types of descriptors to obtain a more comprehensive characterization of the samples.

### 3.1. Low-Level Data Fusion

In this analysis, BA and elemental composition datasets were joined by row-wise matrix augmentation, in which each row corresponded to a replicate of a given sample (it should be noted that samples were analyzed in triplicate), and each column was associated with a compositional variable (i.e., BA or element). The contents of BAs and target elements differed in both the magnitude and amplitude of concentrations, so data autoscaling was applied to equalize their influence in the models.

**Figure 1:**
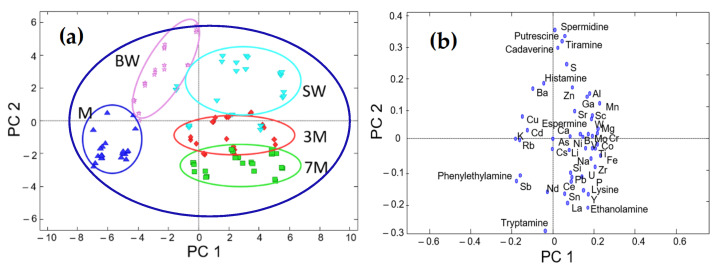
Plots of scores (**a**) and loadings (**b**) from the low-level data fusion. (**a**) Scatter plot of PC1 vs. PC2 scores; (**b**) scatter plot of PC1 vs. PC2 loadings. Sample assignment: M = must (blue); BW = base wine (light purple); SW = stabilized wine (light blue); 3M = sparkling wine with 3 months aging (red); 7M = sparkling wine with 7 months aging (green).

PCA showed a clear structure of samples according to the winemaking stage or process, regardless of other features such as quality or variety (see Figure 1). As a result, the scatter plot of PC1 vs. PC2 scores, which retained more than 43% of data variance, demonstrated clusters according to the sample type, with musts located on the left side, base wines in the upper-left area, stabilized wines predominating in the upper-right quadrant, and sparkling wines mainly in the lower-right part. Sparkling wines were also distinguished based on the aging period, with 3-month-aged wines above 7-month-aged ones. It is important to highlight that this noticeable class separation was not observed when BA and elemental datasets were analyzed separately (see References [33,43]). PC3 retained ca. 15% of the variance, thus providing some additional discrimination patterns (not shown here since the information from 3D plots was more difficult to visualize).

The study of leading descriptors revealed the occurrence of combined or hybrid markers. Elements such as K, Cu, Rb, and Ba were higher in musts compared with the other classes. Some of them (e.g., K and Cu) are the result of agricultural practices such as soil fertilization or mildew treatment. Various BAs—including putrescine, tyramine, spermidine, cadaverine, and histamine—were the dominant features of base and stabilized wines, as the contents of these BAs dramatically increased in the fermentations, especially when malolactic fermentation was applied to reduce the strong malic acidity of wines. A wide range of elements were characteristic of all fermented samples, as their concentrations increased because of oenological treatments with technological additives such as yeasts, tirage liquors, bentonite, and other agents. Among them, Zn, Al, Mn, Fe, Ni, and V can be cited. The BA composition of sparkling wines was different from that of stabilized wines. In this regard, ethanolamine was identified as a marker of sparkling wines since their higher alcoholic degree, achieved through the second fermentation, induced the generation of this compound. As another pattern, BA levels (e.g., cadaverine, histamine, and tyramine) slightly decreased with aging.

As a general conclusion, the BA and elemental compositions of grapes, which were assumed to be similar to the composition of musts, underwent remarkable changes after their transformation in wines. At this step, variations were statistically significant (*p* < 0.05) in all cases. In particular, levels of BAs significantly increased from musts to wines, and then they remained almost constant throughout winemaking processes and barely decayed with aging (except for ethanolamine, which slightly increased with the second fermentation). Similarly, changes in the elemental composition during vinification were dramatic as well due to the set of additives introduced to trigger fermentation and clarification processes.

Thus, an improvement in the characterization and discrimination performance was obtained after applying the data fusion approach.

### 3.2. Mid-Level Data Fusion

As mentioned in the experimental section, the individual matrices of BA and elemental profiles were pretreated by PCA filtering. The scores of three PCs were extracted, as they were a rich source of concentrated information, while irrelevant or ambiguous contributions were excluded from the model.

**Figure 2:**
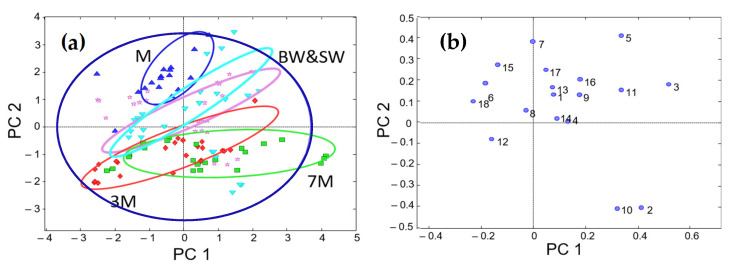
Plots of scores (**a**) and loadings (**b**) from mid-level data fusion. Sample assignment: M = must (blue); BW = base wine (light purple); SW = stabilized wine (light blue); 3M = sparkling wine with 3 months of aging (red); 7M = sparkling wine with 7 months of aging (green). Variable assignment: 1–9 = 1–9 PC of biogenic amines PCA; 10–18 = 1–9 PC of elemental composition PCA.

The row-wise augmented data matrix was evaluated by PCA, and the results obtained are summarized in Figure 2. The scatter plot of PC1 vs. PC2 scores shows a group of musts in the upper-left quadrant, meaning that this class is discriminated from the others. Wines and sparkling wines are scattered throughout the other sectors, without a clear separation among classes but with a certain predominance in some areas. For instance, sparkling wines tend to be across a diagonal (from the bottom left to top right), while base wine samples are located below this area. The other classes are mainly located in intermediate positions. Despite these patterns, the overall performance of this approach was lower than that obtained using the low-level model. On the other hand, the clustering of samples according to quality or variety was not detected either. 

### 3.3. Sample Classification

Supervised studies of sample classification focused on low-level data fusion as, in this particular case, this approach has demonstrated excellent performance in describing the behavior of the samples. Given the natural dependence of sample features with respect to the vinification process, this section investigates the application of the PLS-DA to the classification of samples into the following classes: must, base wine, stabilized wine, 3-month-aged sparkling wine, and 7-month-aged sparkling wine.

**Figure 3:**
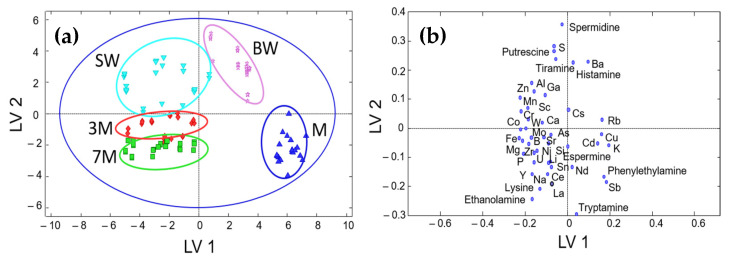
Plots of scores (**a**) and loadings (**b**) from low-level data fusion. (**a**) Scatter plot of LV1 vs. LV2 scores; (**b**) scatter plot of LV1 vs. LV2 loadings. Sample assignment: M = must (blue); BW = base wine (light purple); SW = stabilized wine (light blue); 3M = sparkling wine with 3 months of aging (red); 7M = sparkling wine with 7 months of aging (green).

The first model was established using all of the samples, in which the optimal number of latent variables (LVs) was 3. The plot of scores of LV1 versus LV2 (see Figure 3) shows a remarkable concentration of samples of each class in some specific areas. For example, musts are separated from the others and located in the bottom-right part. The base wines occupy the upper-right part of the graph, also separated from the other classes. Stabilized wines are located in the upper-left quadrant, while sparkling wines are in the lower-left part, showing two groups for 3- and 7-month-aged samples.

For the external validation of the sample classification performance, a set of 120 samples (40 different combinations of class, quality, and variety analyzed in triplicate) was divided into two subsets for training and prediction, with 60 and 40% of the samples randomly selected, respectively. Table 4 summarizes the results using the PLS-DA model using three LVs for the multiclass classification of samples (i.e., classification of each given sample to one of the five classes). The calibration results showed that samples in the must and base wine classes were perfectly classified. For stabilized wine, two samples were incorrectly predicted to be base wine. For sparkling wines, a certain degree of confusion was found between 3- and 7-month-aged samples (two and three samples were misclassified, respectively). For the validation step, no unassigned samples were obtained. Additionally, musts, stabilized wines, and sparkling wines were correctly classified, while a certain amount of confusion was found for base wines (two base wines were classified as stabilized ones). Despite this confusion, which was attributed to the compositional similarities between these two classes, this method opens up promising possibilities for the study of the evolution of oenological samples throughout the vinification process. Furthermore, the performance of classification models based on the integration of BA and elemental profiles was definitively superior to that obtained from the use of each type of data separately.

## 4. Conclusions

In previous papers, it was found that the study and interpretation of descriptive models using biogenic amine or elemental profiles separately provided an incomplete depiction of the evolution of oenological samples throughout the production process of sparkling wines. In this study, this issue was fully solved by combining both sources of information via data fusion. The descriptive performance of a low-level approach using concentrations of BAs and metals was, in this case, superior to that of the mid-level counterpart using PCA scores as the fused data. The oenological process was found to be the principal factor affecting the composition of the studied analytes, while, in this set of samples, quality and variety issues had a lower influence on the description. In particular, excellent discrimination of musts, base wines, stabilized wines, and sparkling wines was realized, thus suggesting that this data combination can be used for successful sample characterization. Some increases in markers of the different classes were identified as well. For instance, high levels of lysine, K, and Cu were detected in musts, while BAs such as putrescine, tyramine, and histamine and elements such as Zn, Al, Mn, and Fe were predominant in base and stabilized wines, and ethanolamine was identified as a biomarker of sparkling wines. Furthermore, PLS-DA successfully classified samples as musts, base wines, stabilized wines, and sparkling wines, meaning that this method can be applied to accurately follow the evolution of oenological samples throughout the winemaking process.

## Figures and Tables

**Table 1:** Recent examples illustrating the potential role of BAs as quality markers.

Analytes	Sample Type	Method	Remarks	Ref.
Putrescine, ethanolamine, histamine, tyramine, cadaverine, phenylethylamine, agmatine, tryptamine, spermine, and spermidine	Musts, base wines, and sparkling wine; Xarel·lo and Pinot Noir varieties	HPLC-FLD with precolumn derivatization using dansyl-Cl	Putrescine, ethanolamine, tyramine, and histamine are the most important in wine quality	[33]
Isopenthylamine, ethanolamine, methylamine, ethylamine, spermidine, spermine, putrescine, tyramine, histamine, cadaverine, and tryptamine	Red and white Croatian wines from Hrvatsko zagorje and Dalmatia regions	HPLC-UV with precolumn derivatization using dansyl-Cl	BAs are a discriminating factor for a grape variety and geographical origin for red wines	[34]
Putrescine, histamine, tyramine, cadaverine, phenylethylamine, tryptamine, spermine, and spermidine	Red and white wines from Chinese markets	HPLC-FLD with precolumn derivatization using dansyl-Cl; liquid–liquid extraction of derivatives	Predominant BAs were putrescine, tyramine, and 2-phenylethylamine	[35]
Putrescine, ethanolamine, histamine, tyramine, cadaverine, phenylethylamine, tryptamine, and agmatine	Red Spanish wines; monovarietal ‘Tempranillo’ wines (young, oak, and aged)	UHPLC-FLD with precolumn derivatization using OPA	Storage time, temperature, and bottle closing influence BA levels. Cork stopper and refrigeration are the best conditions to prevent the increase in histamine and tyramine	[36]
Volatile compounds, amino acids, and amines; agmatine, histamine, spermidine, tyrosine, phenylethylamine, isoamylamine, putrescine, tyramine, and tryptamine	Spanish Sparkling wines from Verdejo, Viura, Malvasia, Albarin, Godello, Prieto Picudo, and Garnacha; “Champenoise” method	HPLC-UV with precolumn derivatization using diethyl ethoxymethylenemalonate	Albarin and Prieto Picudo wines showed the highest BA content	[37]
Methylamine, ethylamine, putrescine, cadaverine, histamine, spermidine, spermine, phenylethylamine, tyramine, and tryptamine	Alcoholic beverages including red and white wine	Ion-pair chromatography with butane-sulfonic acid; HPLC-potentiometric detection; multi-walled carbon nanotube sensing membrane	Tyramine and tryptamine are the most abundant in red wine; spermidine, spermine, and tryptamine are the most abundant in white wine	[38]
Histamine, putrescine, cadaverine, and tyramine	“Refosk” wine from Slovenian-Italian Karst region	HPLC-UV with precolumn derivatization using dansyl-Cl	Some strains of *Lactobacillus* have the ability to produce BAs	[39]
Cadaverine, hexylamine, histamine, phenylethylamine, putrescine, and tyramine	Chinese wines	Direct separation and detection by UHPLC-QqQ-MS/MS; QuEChERS for sample treatment	Histidine is correlated with alcoholic degree and grape variety; phenylethylamine is correlated with pH and storage time	[40]

**Table 2:** List of samples under study. Sample codes are as follows: M, must; BW, base wine; SW, stabilized wine; C3, 3 months in rhyme cava wine (sparkling wine); C7, 7 months in rhyme cava wine (sparkling wine); P, Pinot Noir; X, Xarel·lo; A, quality A; B, quality B; C, quality C; D, quality D (reproduced from Ref. [33]).

Grape Variety	Quality	Must	Base Wine	Stabilized Wine	3-Month Sparkling Wine	7-Month Sparkling Wine
Pinot Noir	A	MPA	BWPA	SWPA	C3PA	C7PA
	B	MPB	BWPB	SWPB	C3PB	C7PB
	C	MPC	BWPC	SWPC	C3PC	C7PC
	D	MPD	BWPD	SWPD	C3PD	C7PD
Xarel·lo	A	MXA	BWXA	SWXA	C3XA	C7XA
	B	MXB	BWXB	SWXB	C3XB	C7XB
	C	MXC	BWXC	SWXC	C3XC	C7XC
	D	MXD	BWXD	SWXD	C3XD	C7XD

**Table 3:** Determination of various relevant compounds in the different samples. Concentrations are expressed in mg L^−1^. Bold numbers denote samples with higher values.

Sample	Ethanolamine	Putrescine	Histamine	S	K	Na
MPA	2.99	2.52	0.16	2.68	**93.3**	1.22
MPB	2.70	1.42	0.14	5.86	**151.0**	2.77
MPC	3.85	4.84	0.17	8.04	**124.1**	2.05
MPD	3.49	2.14	0.13	3.83	**151.3**	2.59
MXA	2.72	1.29	0.11	3.48	**72.9**	1.87
MXB	4.01	0.43	0.10	2.91	**87.6**	2.16
MXC	5.30	3.29	0.11	3.46	**120.8**	1.78
MXD	4.09	2.81	0.12	3.31	**95.2**	1.40
BWPA	3.14	4.01	0.19	**32.5**	47.4	0.59
BWPB	5.21	3.42	0.18	**33.9**	79.0	0.50
BWPC	5.35	**24.10**	**4.00**	**32.7**	96.6	2.02
BWPD	6.13	**21.43**	**3.68**	**22.5**	77.5	3.38
BWXA	3.86	1.81	0.11	**17.7**	38.8	0.50
BWXB	5.14	3.05	0.11	**43.0**	78.9	1.37
BWXC	5.75	**10.79**	**1.76**	**57.9**	63.7	3.27
BWXD	6.51	**13.07**	**1.94**	**40.9**	75.7	2.44
SWPA	3.43	3.77	0.20	**37.5**	34.6	1.12
SWPB	5.49	2.80	0.31	**25.9**	37.6	2.23
SWPC	4.75	**10.81**	**1.20**	**24.3**	46.0	2.82
SWPD	6.57	**15.87**	**1.78**	**16.4**	30.4	5.18
SWXA	3.22	0.95	0.11	**16.9**	34.0	0.70
SWXB	6.29	2.43	0.21	**22.7**	27.2	2.00
SWXC	5.94	**14.37**	**2.32**	**21.6**	28.8	3.98
SWXD	7.13	**10.26**	**1.77**	**21.5**	35.7	4.63
C3PA	2.89	2.11	0.18	14.2	26.2	2.48
C3PB	6.05	2.69	0.30	25.9	37.1	2.28
C3PC	4.94	**12.00**	**1.40**	20.8	44.7	3.43
C3PD	6.42	**15.90**	**2.32**	16.8	25.4	4.69
C3XA	3.41	1.11	0.13	11.7	30.7	2.05
C3XB	7.14	3.14	0.26	24.4	14.2	2.40
C3XC	7.25	**17.83**	**2.64**	21.2	25.9	5.23
C3XD	6.08	**9.47**	**1.69**	19.9	39.3	5.16
C7PA	2.73	1.39	0.14	14.5	30.6	2.42
C7PB	5.50	2.28	0.26	25.3	40.7	2.20
C7PC	5.04	**11.47**	**1.38**	21.5	45.5	3.46
C7PD	6.74	**18.47**	**2.90**	19.1	21.8	5.70
C7XA	3.44	0.94	0.12	12.2	32.4	1.99
C7XB	5.55	3.11	0.26	23.4	30.4	3.42
C7XC	5.88	**10.84**	**1.87**	20.8	39.7	5.41
C7XD	6.06	**18.50**	**2.79**	21.2	41.0	5.04

**Table 4:** Summary of classification results with the percentages of correctly classified samples in both calibration and validation steps using PLS-DA.

Classification Rate					
Step	Must	Base Wine	Stabilized Wine	3-Month Sparkling Wine	7-Month Sparkling Wine
Calibration	100%	100%	90% ^1^	87% ^2^	75% ^3^
Validation	100%	70% ^4^	100%	100%	100%

Misclassifications are as follows: ^1^ predicted as base wine; ^2^ predicted as 7-month-aged sparkling wine; ^3^ predicted as 3-month-aged sparkling wine; ^4^ predicted as stabilized wine.

## Data Availability

Data are contained within the article.
